# Development and validation of a 16-gene T-cell- related prognostic model in non-small cell lung cancer

**DOI:** 10.3389/fimmu.2025.1566597

**Published:** 2025-04-07

**Authors:** Anbing Zhang, Huang Ting, Jun Ma, Xiuqiong Xia, Xiaoli Lao, Siqi Li, Jianping Liang

**Affiliations:** ^1^ Department of Pulmonary and Critical Care Medicine, Zhongshan People’s Hospital, Zhongshan, China; ^2^ Shenzhen University Medical School, Shenzhen University, Shenzhen, China; ^3^ Graduate School, Guangdong Medical University, Zhanjiang, China

**Keywords:** non-small cell lung cancer, T lymphocyte, prognosis, tumor immune microenvironment, bioinformatics

## Abstract

**Background:**

Non-small cell lung cancer (NSCLC) exhibits variable T-cell responses, influencing prognosis and outcomes.

**Methods:**

We analyzed 1,027 NSCLC and 108 non-cancerous samples from TCGA using ssGSEA, WGCNA, and differential expression analysis to identify T-cell-related subtypes. A prognostic model was constructed using LASSO Cox regression and externally validated with GEO datasets (GSE50081, GSE31210, GSE30219). Immune cell infiltration and drug sensitivity were assessed. Gene expression alterations were validated in NSCLC tissues using qRT-PCR.

**Results:**

A 16-gene prognostic model (LATS2, LDHA, CKAP4, COBL, DSG2, MAPK4, AKAP12, HLF, CD69, BAIAP2L2, FSTL3, CXCL13, PTX3, SMO, KREMEN2, HOXC10) was established based on their strong association with T-cell activity and NSCLC prognosis. The model effectively stratified patients into high- and low-risk groups with significant survival differences, demonstrating strong predictive performance (AUCs of 0.68, 0.72, and 0.69 for 1-, 3-, and 5-year survival in the training cohort). External validation confirmed its robustness. A nomogram combining risk scores and clinical factors improved survival prediction (AUCs>0.6). High-risk patients responded better to AZD5991-1720, an MCL1 inhibitor, while low-risk patients showed improved responses to IGF1R-3801-1738, an IGF1R inhibitor, suggesting that risk stratification may help optimize treatment selection based on tumor-specific vulnerabilities. qRT-PCR validation confirmed the differential expression of model genes in NSCLC tissues, consistent with TCGA data.

**Conclusion:**

We identified a 16-gene T-cell-related prognostic model for NSCLC, which stratifies patients by risk and predicts treatment response, aiding personalized therapy decisions. However, prospective validation is needed to confirm its clinical applicability. Potential limitations such as sample size and generalizability should be considered.

## Introduction

Non-small cell lung cancer (NSCLC) remains the leading cause of cancer-related mortality worldwide, accounting for approximately 85% of all lung cancer cases (1, 2). Lung adenocarcinoma (LUAD) and lung squamous cell carcinoma (LUSC) are the major subtypes of NSCLC, constituting about 40% and 30% of cases, respectively (3). The prognosis of NSCLC varies significantly, with a general 5-year survival rate of around 24%, which decreases to about 6% in advanced stages (4). Compared to other malignancies, such as breast cancer (approximately 90%) (5) and prostate cancer (nearly 100%) (6), NSCLC has markedly lower survival rates, underscoring the urgent need for improved prognostic tools. Developing effective prognostic tools is crucial for enabling personalized treatment approaches, which can improve patient survival and quality of life. Current research focuses on molecular signatures, such as gene mutations (7), protein expression (8), and RNA profiles (9), as potential prognostic biomarkers. However, these biomarkers often fail to fully capture the influence of the tumor immune microenvironment, particularly T-cell activity, which plays a crucial role in tumor progression and response to therapy (10). The tumor immune landscape, especially T-cell infiltration and function, is highly heterogeneous and poorly reflected in existing molecular classifiers, limiting their clinical predictive power (11). Furthermore, the clinical translation of current prognostic markers remains challenging due to the heterogeneity of the disease and variable patient responses to treatment (12). Therefore, more reliable biomarkers that integrate immune-related parameters are needed to improve prognosis prediction and guide personalized treatment strategies.

Recent research on NSCLC has increasingly focused on the role of the immune system, particularly T-cells, in influencing disease progression and treatment outcomes (13). The effect of T-cells in NSCLC is influenced by various T-cell-related genes, which regulate T-cell activation, proliferation, and survival (14, 15). These genes include those encoding cytokines, checkpoint proteins, and other molecules involved in immune response (16–18). Current immunotherapies, particularly immune checkpoint inhibitors (e.g., PD-1/PD-L1 and CTLA-4 inhibitors), enhance T-cell-mediated antitumor activity by blocking the pathways cancer cells use to evade immune detection (19, 20). Given the critical role of T-cells in NSCLC, T-cell-related gene expression pattern have the potential to serve as prognostic markers, helping to optimize treatment strategies and improve patient stratification. Although studies have demonstrated the prognostic significance of tumor-infiltrating T-cells, including CD4+, CD8+, and FOXP3+ subsets, in NSCLC (21), these findings have primarily relied on immunohistochemical assessments, which do not fully capture the complexity of T-cell-related gene expression and its prognostic implications. Consequently, integrating these immune markers into computational prognostic models remains limited. To address this gap, reliable biomarkers based on transcriptomic profiling of T-cell-related genes are needed to enhance prognosis prediction and guide personalized treatment strategies in NSCLC.

In this study, we analyzed NSCLC transcriptomic data from public databases using weighted gene co-expression network analysis (WGCNA), single-sample gene set enrichment analysis (ssGSEA), and least absolute shrinkage and selection operator (LASSO) Cox to identify key T-cell-related genes and assess their impact on patient prognosis. WGCNA (22) was employed to identify co-expressed gene modules associated with T-cell abundance, as determined by ssGSEA, uncovering T-cell-related gene networks that influence NSCLC progression. ssGSEA quantifies immune-related gene expression at the individual sample level (23), capturing inter-patient variability in immune infiltration. LASSO Cox regression, an extension of the Cox proportional hazards model, was used to construct a prognostic model by selecting the most relevant genes while minimizing overfitting (24). Additionally, we explored the interactions between these genes and the tumor immune microenvironment to develop a robust T cell-related prognostic model. The objective was to identify specific T cell-related genes and their expression patterns in NSCLC that could serve as effective prognostic markers and guide more personalized immunotherapy strategies.

## Materials and methods

### Data source and processing

Transcriptomic data of the LUAD and LUAC cohorts were obtained from The Cancer Genome Atlas (TCGA) via the UCSC Xena Browser. Each dataset included cancerous and adjacent non-cancerous tissue samples with expression levels originally quantified in Fragments Per Kilobase Million (FPKM). To standardize expression levels across samples, the FPKM values were transformed into Transcripts Per Million (TPM) using the formula:


$$TPM_{i}=(\frac{FPKM_{i}}{\sum_{j}FPKM_{j}}\cdot 10^{6})$$

After transformation, expression profiles from different sources were merged, and batch effects arising from technical variability were corrected using the ComBat function from the sva package. ComBat was chosen for its ability to mitigate systematic biases while preserving true biological variability, ensuring comparability between datasets. Additionally, transcriptome data from GEO datasets (GSE50081, GSE31210, and GSE30219) were integrated for validation. To maintain consistency in gene expression representation, the median expression value was used when multiple expression values existed for the same gene. These GEO datasets complement TCGA data by introducing diverse patient cohorts, thereby enhancing the robustness and generalizability of the prognostic model. Overall survival time was uniformly recorded in days across all datasets, ensuring consistency in survival analysis. A summary of sample information is provided in **Table 1**.

**Table 1:** Dataset information.

ID	Platform	Sample type	N (cancerous: non-cancerous tissues)	Data type
TCGA		cancerous and adjacent non-cancerous tissues	1135 (1027: 108)	RNAseq
GSE50081	GPL570	cancerous tissue	173	mRNA array
GSE31210	GPL570	cancerous tissue	225	mRNA array
GSE30219	GPL570	cancerous tissue	190	mRNA array

### ssGSEA

The GSVA package in R was used to perform single-sample Gene Set Enrichment Analysis (ssGSEA) to calculate the enrichment scores of 28 immune cell subtypes in the TCGA cohorts (25). ssGSEA allows for the transformation of gene expression matrices into scores that reflect the degree of immune cell presence in each sample. A previous study applied ssGSEA to define immune cell infiltration clusters and develop an immune-related prognostic model in osteosarcoma, supporting its reliability in cancer prognosis (26). In this study, the ssGSEA-derived immune cell abundance scores were utilized as phenotypic traits in WGCNA to identify T-cell-related hub genes critical for NSCLC progression. These scores also enabled the evaluation of tumor immune microenvironment differences between risk groups, contributing to the development of a prognostic model and the assessment of immunotherapy response potential. Samples with p-values less than 0.05 were included in further analyses, and immune cell types with zero abundance across all samples were excluded.

### WGCNA

WGCNA was conducted using the WGCNA package to identify T-cell-related genes, where immune cell abundances served as phenotype traits. WGCNA helps detect clusters (modules) of co-expressed genes and examines their associations with specific traits, making it ideal for identifying genes linked to T-cell activity. Outliers were removed by hierarchical clustering, and an optimal soft threshold, a power parameter that enhances strong gene-gene correlations while reducing noise, was determined to ensure the network’s scale-free topology. Modules were assigned unique colors and were further screened based on their correlation with phenotype. Modules with a correlation coefficient ≥ 0.5 were selected for further analysis (27). Hub genes within each module were identified based on gene significance (GS) and module membership (MM) values. The thresholds |GS| > 0.2 and |MM| > 0.8 indicate strong associations by ensuring selected genes are significantly correlated with the phenotype and highly connected within their module (28). T-cell-related genes were identified through correlation analysis between gene expression and T-cell abundance. Genes with a high correlation coefficient (|correlation| > 0.5) and a significant p-value (< 0.05) were considered T-cell-related genes and were then intersected with the hub genes to identify T-cell-related hub genes. A correlation threshold of 0.5 was selected to capture genes with a moderate to strong association (29) with T-cell levels while avoiding overly restrictive cutoffs that might exclude biologically relevant genes. The p-value threshold of < 0.05 ensures statistical significance while maintaining a sufficient gene pool for downstream analysis.

### Enrichment analysis

Functional enrichment analysis on T-cell-related hub genes was performed using the ClusterProfiler package in R. This package facilitates the identification of overrepresented Gene Ontology (GO) and Kyoto Encyclopedia of Genes and Genomes (KEGG) pathways, providing insights into the biological processes and pathways associated with these genes. P-values were adjusted using the Benjamini-Hochberg method to control for false discovery rates, ensuring robust results based on adjusted p-values.

### Identification of T-cell-related differentially expressed genes

DEGs in NSCLC were identified using the Limma package, which is widely used for its ability to handle multiple comparisons and model gene expression differences effectively. Genes with |logFC| > 0.5 and adjusted p-value < 0.05 were considered DEGs (30). These genes were then cross-referenced with T-cell-related hub genes to identify T-cell-related DEGs within the NSCLC datasets.

### Consensus clustering

To classify T-cell-related NSCLC subtypes, consensus clustering was performed using the ConsensusClusterPlus package. This approach aggregates results from multiple iterations, ensuring robust and stable clustering of samples based on the expression profiles of T-cell-related DEGs. Euclidean distance was used for clustering, with 50 iterations and 80% subsampling per iteration. Parameters such as reps = 50, pItem = 0.8, and distance = Pearson were set to enhance clustering reliability. The optimal number of subtypes (k) was determined by evaluating the Cumulative Distribution Function (CDF) curve, which measures the relative change in area under the CDF curve, ensuring a balance between stability and granularity. Additionally, consensus heatmaps were examined for clear boundaries between clusters, and delta area plots were used to assess the improvement in clustering stability with increasing k values. The optimal k was selected based on minimal fluctuations in the CDF curve and relatively higher consensus scores, indicating robust cluster separation. DEGs between these subtypes were further identified using the Limma package, considering genes with |logFC| > 1 and adjusted p-value < 0.05 as inter-subtype DEGs (31).

### Gene set variation analysis

Differential analysis of GO and KEGG pathways across T-cell-related NSCLC subtypes was conducted using the GSVA package. GSVA enables a non-parametric, unsupervised assessment of gene set variation, which is ideal for comparing pathway activities across subtypes. Pathways with an adjusted p-value < 0.05 were considered significantly different.

### Construction of a prognostic gene model

Patients from the TCGA cohorts were randomly divided into training and testing sets in a 7:3 ratio. The glmnet package was employed to conduct LASSO Cox regression analysis on the expression profiles of inter-subtype DEGs within the training set. Unlike traditional univariate or multivariate Cox regression models, LASSO Cox regression employs L1 regularization to address multicollinearity, automatically selecting the most relevant genes and improving model stability. This feature is particularly beneficial when working with high-dimensional datasets, such as gene expression data, by reducing the risk of overfitting and enhancing predictive accuracy (32). The optimal penalty parameter λ was determined based on the minimum criterion, and genes with non-zero coefficients were retained as optimal variables. The risk score was calculated using the weight coefficients and gene expression levels, providing a quantifiable measure for prognostic evaluation (33):


$$RiskScore\;=\sum_{\text{i}}^{n}coefi\times genei$$

### Protein-protein interaction networks

To explore the interactions among model genes and other genes with similar functions, PPI networks were constructed using GeneMANIA (http://www.genemania.org). GeneMANIA is a robust tool that integrates data from multiple sources, allowing for the visualization of gene-gene interactions, including physical interactions, co-expression, and shared pathways. This helps in identifying potential functional relationships and interactions that could provide deeper insights into the biological roles of the model genes.

### Nomogram construction and evaluation

Univariate and multivariate Cox analyses were conducted on risk scores and clinical variables such as age, smoking history, and tumor stage to identify independent prognostic factors. The results were visualized using forest plots generated by the “forestplot” package in R, which clearly displays the hazard ratios and confidence intervals, highlighting which factors independently affect prognosis. A nomogram was constructed using the “rms” package to combine these independent prognostic factors, providing a user-friendly graphical tool for predicting individual patient outcomes. The nomogram was assessed through calibration curves (using the calibrate function from “rms”), which compare predicted versus observed outcomes.

### Prediction of immunotherapy response

To estimate how patients in the TCGA cohorts would respond to anti-PD-1/PD-L1 and anti-CTLA4 immunotherapy, the Tumor Immune Dysfunction and Exclusion (TIDE) tool (http://tide.dfci.harvard.edu) was used. TIDE is a computational method that predicts immune evasion mechanisms, helping to assess which patients are more likely to benefit from specific immunotherapy treatments. This can be critical in personalizing treatment strategies for patients based on their predicted response.

### GSEA

To identify enriched Hallmark pathways in high-risk patients, GSEA was conducted on the Hallmark gene set using the “clusterProfiler” package in R. GSEA helps in identifying whether a predefined set of genes shows statistically significant, concordant differences between two biological states, providing insights into the molecular pathways that are active in high-risk versus low-risk groups. Pathways with an adjusted p-value < 0.05 were considered significant, indicating their potential relevance in the disease context.

### Drug sensitivity prediction

Drug sensitivity was assessed using the calcPhenotype function from the “oncoPredict” package. Gene expression matrices and drug treatment information from the Genomics of Drug Sensitivity in Cancer (GDSC2) and CTRP V2 databases (https://osf.io/c6tfx/). The “oncoPredict” package allowed for the prediction of drug response by modeling IC50 values for the TCGA cohorts based on expression profiles. This analysis aids in identifying which patients might respond better to certain drugs, thus guiding more personalized therapy choices. The correlation between IC50 values and risk scores was then analyzed to identify potential drug candidates for different risk groups.

### Quantitative real-time polymerase chain reaction

The study included eight lung adenocarcinoma samples, which were obtained from tumor tissues and adjacent paracancerous tissues of hospitalized patients at Zhongshan People’s Hospital. Participants were selected based on the following inclusion criteria: individuals aged 18 to 85 years with a confirmed pathological diagnosis of lung adenocarcinoma, who had not undergone any immunotherapy, chemotherapy, or radiotherapy before enrollment. Additionally, participants needed to have good organ function and be free of significant complications or chronic illnesses. The exclusion criteria ruled out patients with severe immune deficiencies (such as HIV infection or those undergoing immunosuppressive therapy), individuals with autoimmune conditions, organ transplant recipients, as well as pregnant or breastfeeding women. All patients were required to sign an informed consent form prior to enrollment, confirming their understanding of the study’s purpose and potential risks. Total RNA was extracted from lung cancer tissue and adjacent paracancerous tissue samples using Trizol reagent (Invitrogen, USA). Reverse transcription was performed using PrimeScript™ RT reagent Kit (TAKARA, RR047A), and cDNA amplification was carried out using SYBR^®^ Premix Ex TaqTM II (TAKARA, RR820A) on an Applied Biosystems™ 7500 (Thermo Fisher, Singapore, 4351104). Primer sequences used are summarized in **Supplementary Table S1**, and GAPDH was used as an internal reference. The relative expression of the target genes was calculated by the 2^-ΔΔCt^ method, providing a quantifiable measure of gene expression differences between cancerous and adjacent paracancerous tissues.

### Statistical analysis

Statistical analyses were conducted using R (v4.3.1). The distribution of T-cell subsets in different risk groups was visualized using a t-distributed stochastic neighbor embedding (t-SNE) analysis. The R packages “survival” and “survminer” were employed for survival analysis and visualization, while the “timeROC” package was used for time-dependent receiver operating characteristic (ROC analysis. Heatmaps were visualized using the “pheatmap” package. ROC curves were generated using the “pROC” package. Differential expression analysis was performed using the Limma package with an empirical Bayes approach, applying a threshold of |logFC| > 0.5 and adjusted p-value < 0.05. Survival analysis was conducted using Kaplan-Meier estimates with the log-rank test. LASSO Cox regression was used for prognostic modeling, with λ optimized through cross-validation to prevent overfitting. Univariate and multivariate Cox regression identified independent prognostic factors, with hazard ratios and confidence intervals visualized using forest plots. For correlation analysis, Pearson correlation was applied under the assumption of linear relationships, and Wilcoxon rank-sum tests were used where normality was not assumed. t-tests were performed for differential analysis between groups. Model performance was assessed using time-dependent ROC curves and calibration plots to evaluate predictive accuracy and clinical utility. Statistical significance was set at p < 0.05. Unless otherwise specified, all results were visualized using “ggplot2” or “plot”.

## Results

### Identification of T-cell-related hub genes in NSCLC

**Figure 1:**
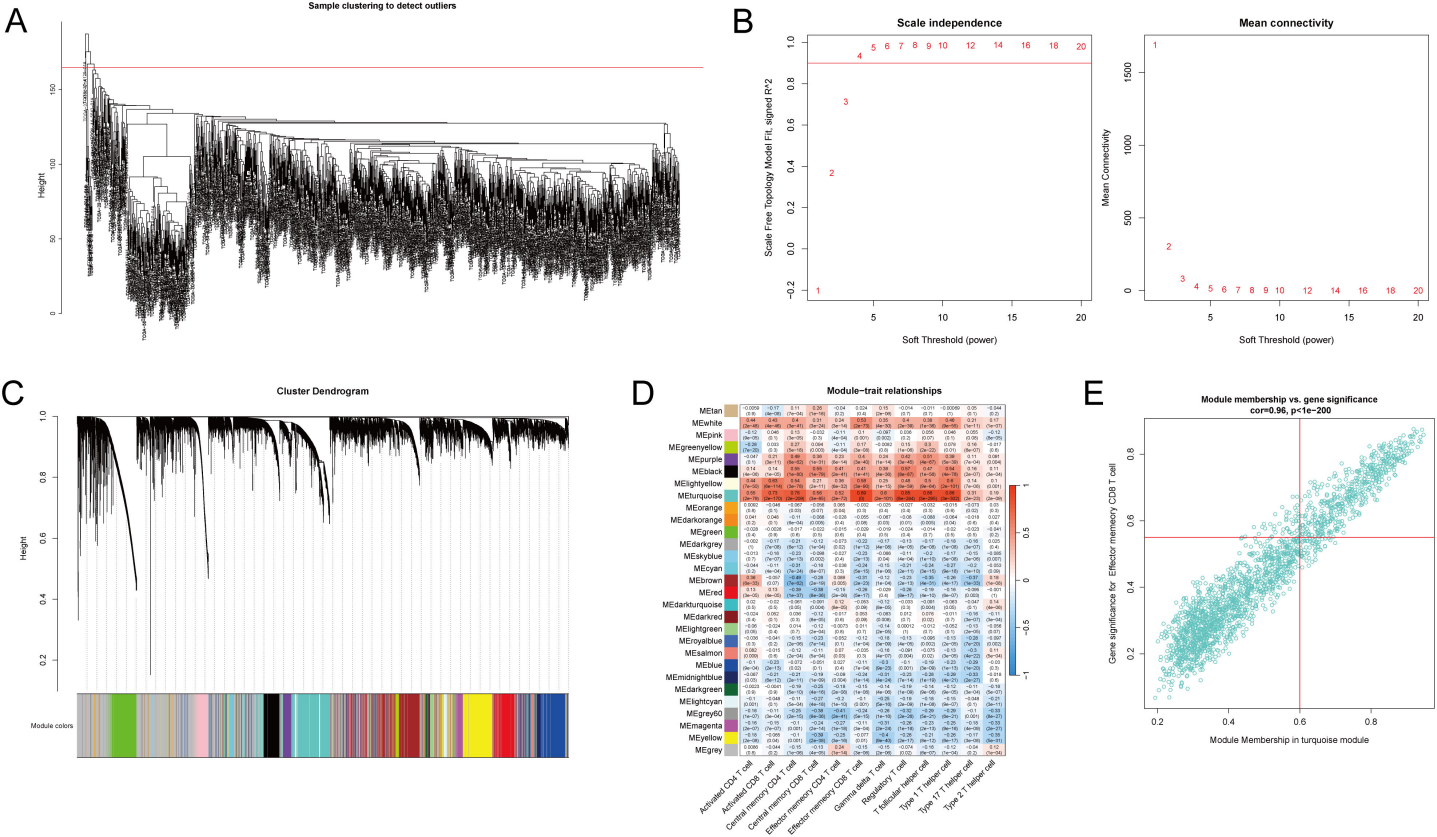
Identification of T-cell-related hub genes in non-small cell lung cancer (NSCLC) patients. The transcriptomic data of the lung adenocarcinoma (LUAD) and lung squamous cell carcinoma (LUAC) cohorts from The Cancer Genome Atlas (TCGA) were acquired, consisting of 1027 cancer tissue samples and 108 non-cancerous tissue samples. ssGSEA and WGCNA were performed on 1027 cancer tissue samples to identify T-cell-related hub genes in NSCLC patients. **(A)** Quality control and preprocessing of TCGA cancer tissue samples. Two outliers were excluded. **(B)** Determination of the soft-thresholding power for WGCNA set at 0.9 to ensure a scale-free network. **(C)** Visualization of the gene clustering process and the identification of co-expression modules, with colors indicating the distinct modules. **(D)** The enrichment scores of 28 immune cell subtypes were estimated via ssGSEA. Heatmap represents the correlation between module eigengenes and immune cell enrichment scores, with the color intensity reflecting the strength of correlation. Five modules (turquoise, light-yellow, black, purple, and white) with a correlation coefficient ≥ 0.5 with the phenotype were identified as core modules. **(E)** T-cell-related genes were identified through correlation analysis between gene expression and T-cell abundance. Genes with |gene significance (GS)| > 0.2 and |module membership (MM)| > 0.8 were considered hub genes. A scatter plot illustrates the correlation between GS related to effector memory CD8 T-cells and MM in the turquoise module in NSCLC samples.

To identify T-cell-related hub genes in NSCLC, we performed ssGSEA and WGCNA on 1027 cancer tissue samples from the TCGA NSCLC cohorts. We computed enrichment scores of 28 immune cell subtypes for each sample using ssGSEA, using these scores as phenotypic data in WGCNA. Two outliers were identified and excluded based on hierarchical clustering and standardized connectivity scores, ensuring that extreme data points did not distort network construction (**Figure 1A**). A soft threshold of 0.9 was applied to establish a scale-free network topology (**Figure 1B**). A total of 29 modules were identified (**Figure 1C**). Among them, five modules with a correlation coefficient ≥ 0.5 with the phenotype were selected for further analysis, including the turquoise, light yellow, black, purple, and white modules (**Figure 1D**). Hub genes within these modules were identified based on the criteria of |GS| > 0.2 and |MM| > 0.8. Additionally, genes showing |correlation| > 0.5 with T-cell abundance and a p-value < 0.05 were classified as T-cell-related genes (**Figure 1E**). These genes were then intersected with the hub genes to determine T-cell-related hub genes associated with NSCLC.

### Identification and characterization of T-cell-related DEGs in NSCLC

**Figure 2:**
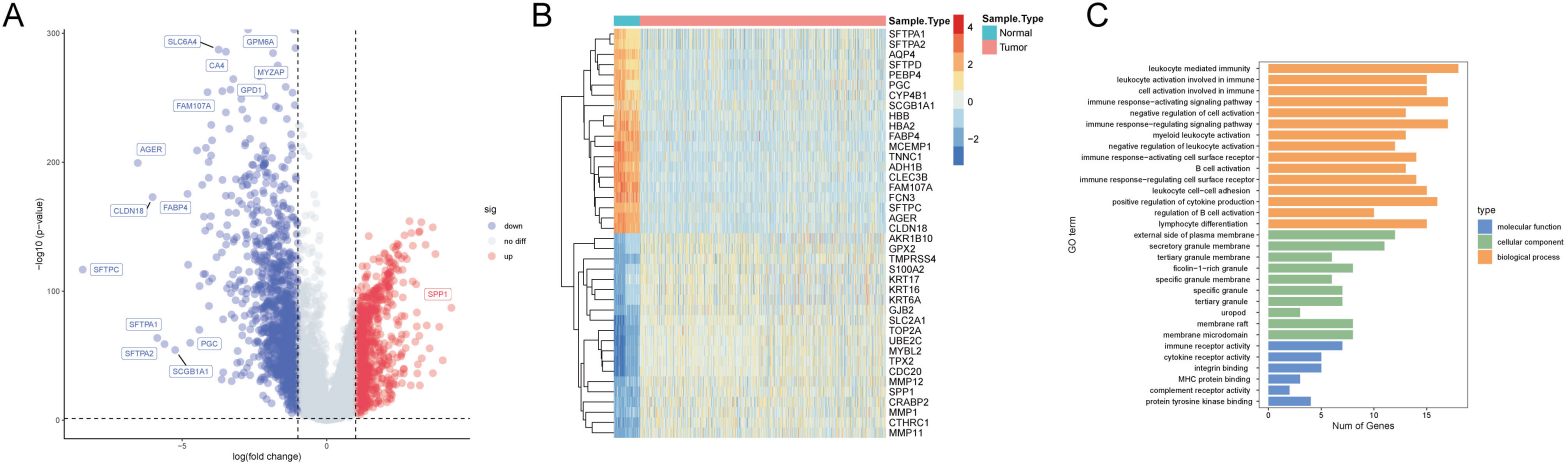
Transcriptomic and Gene Ontology (GO) analysis in the TCGA NSCLC cohorts. **(A)** A volcano plot illustrates the differential expression between cancer and non-cancerous tissue samples, with upregulated genes in red, downregulated genes in blue, and non-significant genes in grey. Key significantly altered genes are labeled. **(B)** A heatmap displays the expression patterns of the top 40 genes in each sample. Expression levels are color-coded from blue (low expression) to red (high expression), distinguishing between non-cancerous (blue bar) and cancer (orange bar) samples. **(C)** The GO enrichment analysis results are categorized by biological process (orange), cellular component (green), and molecular function (blue), with the number of genes represented on the x-axis and the enriched GO terms on the y-axis.

To identify T-cell-related DEGs associated with NSCLC, we analyzed 1027 cancer tissue samples and 108 non-cancerous tissue samples in the TCGA NSCLC cohorts. Differential gene expression analysis revealed 2,063 DEGs (865 upregulated and 1,198 downregulated genes) in the cancer group compared to the control group (**Figure 2A**, **Supplementary Table S2**). Expression profiles of the top 40 DEGs in each sample are shown in **Figure 2B**. Intersecting of T-cell-related hub genes with DEGs identified 80 T-cell-related DEGs. GO enrichment analysis revealed that these DEGs were significantly enriched in 15 biological process terms (e.g., leukocyte-mediated immunity, leukocyte activation involved in immune response, and cell activation involved in immune response), 5 cellular component terms (e.g., plasma membrane external side, secretory granule membrane, and tertiary granule membrane), and 6 molecular function terms (e.g., immune receptor activity, cytokine receptor activity, and integrin binding) (**Figure 2C**, **Supplementary Table S3**).

### Identification of T-cell-related subtypes in NSCLC via consensus clustering

**Figure 3:**
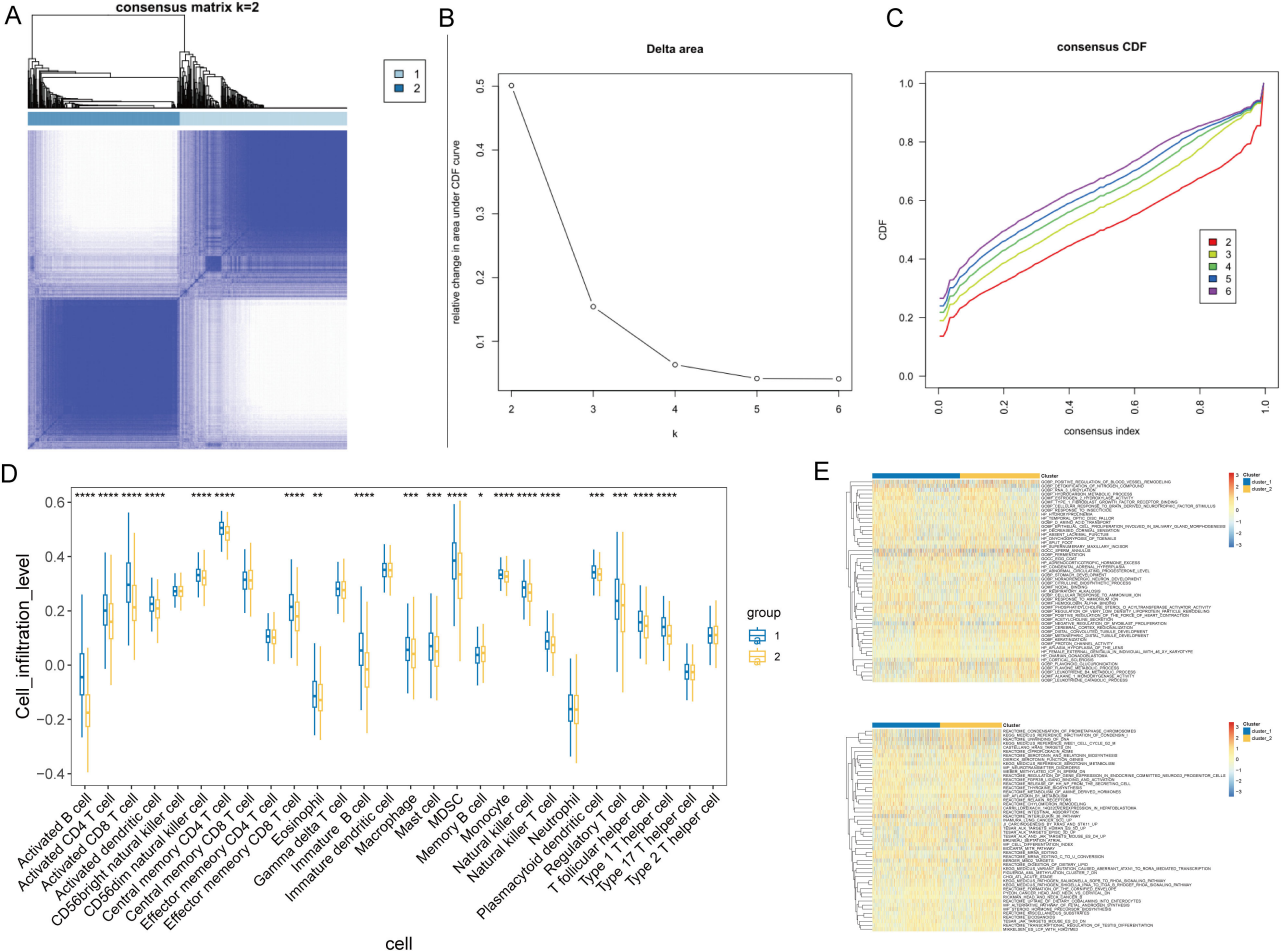
Consensus clustering and immune profile analysis in NSCLC. **(A)** Representative image of a consensus matrix for k = 2. **(B)** Determination of the optimal number of clusters (k) by delta area plot. The lowest delta area corresponds to k = 2. **(C)** Consensus cumulative distribution function (CDF) curves for different numbers of clusters (k = 2 to 6), demonstrating the stability of the two-cluster solution. **(D)** Comparison of the infiltration levels of 20 immune cell types between NSCLC subgroups. *P < 0.05, ^**^ *P* < 0.05, ^***^ *P* < 0.01, ^***^ *P* < 0.001. **(E)** GSVA-based heatmap illustrates the functional enrichment of T-cell-related DEGs across NSCLC subgroups for GO and KEGG pathways. Color intensity represents enrichment scores.

To identify T-cell-related NSCLC subtypes, we performed consensus clustering analysis on the TCGA cohort (n=1,135) using the expression profiles of 80 T-cell-related DEGs. The analysis indicated an optimal division into two subgroups, as demonstrated by minimal fluctuations in the consensus index of the CDF curves and relatively higher consensus scores when patients were clustered into two categories (**Figures 3A–C**). Immune microenvironment analysis of different subtypes showed significant differences in 20 immune cell types between these clusters (**Figure 3D**). Group 1 exhibited significantly higher or trending higher infiltration of most immune cell types, including activated CD8^+^ T cells, central memory CD4^+^ T cells, dendritic cells, and NK cells, suggesting an immune-active phenotype with enhanced antitumor immunity. In contrast, Group 2 showed an increase only in memory B cells, indicating a relatively immune-suppressed or less inflamed tumor microenvironment. Furthermore, GSVA for GO and KEGG pathways revealed differential functional profiles between the patient subtypes (**Figure 3E**, **Supplementary Tables S4**, **S5**).

### Development of a T-cell prognostic gene signature for NSCLC

**Figure 4:**
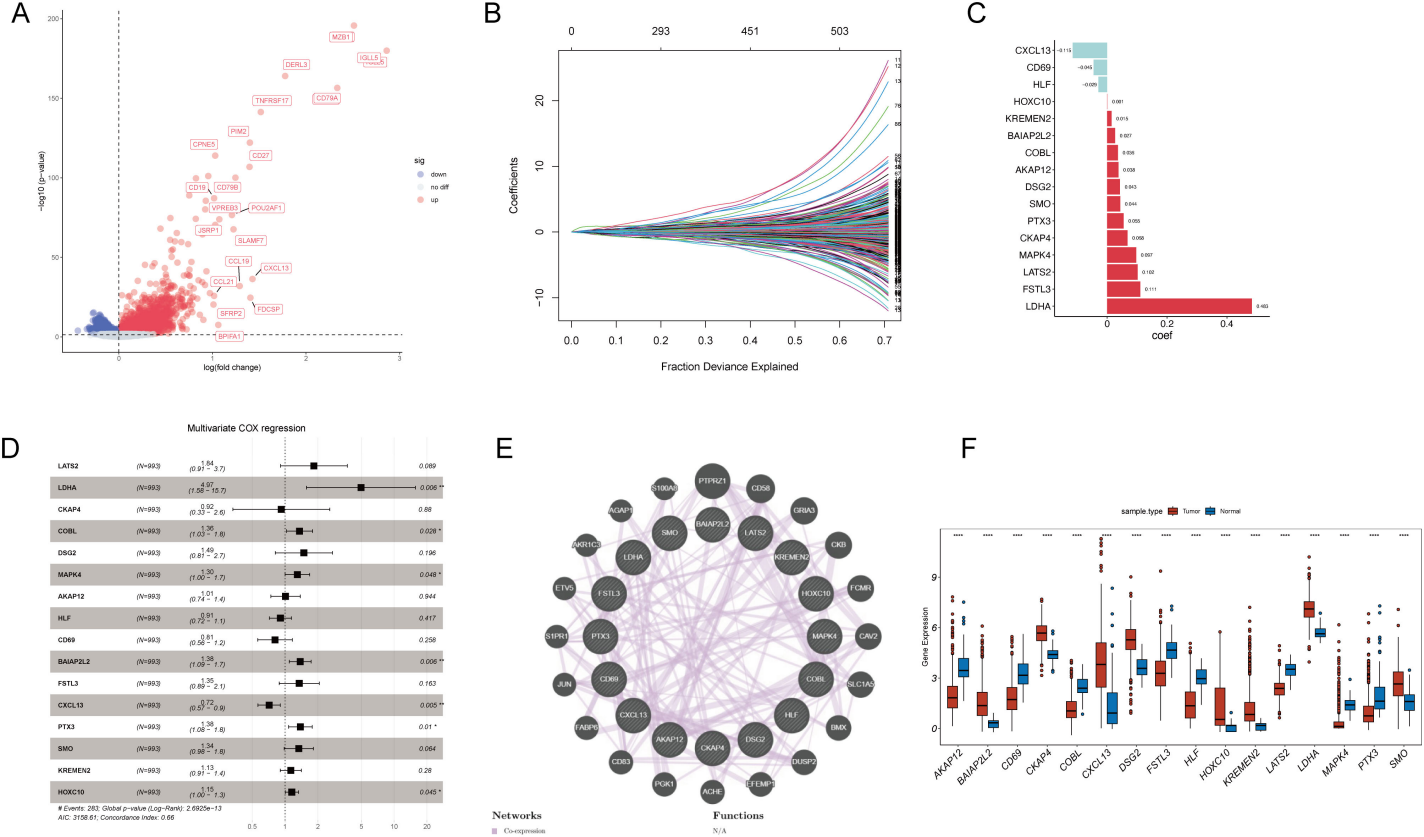
Prognostic model development and validation for T-cell-related genes in NSCLC. **(A)** A volcano plot displays DEGs between T-cell-related NSCLC subtypes, with upregulated genes in red and downregulated genes in blue. **(B)** LASSO Cox regression analysis was conducted to select the most prognostically relevant genes. **(C)** A bar graph presents the corresponding coefficients of the selected genes. **(D)** Multivariable Cox analysis shows hazard ratios and p-values for the selected genes, indicating their prognostic significance. **(E)** Protein-protein interaction network highlights the interactions between proteins encoded by the prognostic genes. **(F)** Boxplots demonstrate the significant differential expression of the prognostic genes between cancer and non-cancerous tissues in NSCLC. *p < 0.05; **p < 0.01; ***p < 0.001; ****p < 0.0001.

To construct a prognostic model based on T-cell-related genes, we initially identified 5,766 DEGs, with 2860 upregulated and 2906 downregulated in Cluster1 compared to Cluster2 (**Figure 4A**). Then, we sought to identify genes associated with prognosis among the inter-subtype DEGs. The TCGA cohorts were randomly divided into a training set and a validation set in a 7:3 ratio. A LASSO Cox regression analysis conducted in the training set identified 16 genes, including LATS2, LDHA, CKAP4, COBL, DSG2, MAPK4, AKAP12, HLF, CD69, BAIAP2L2, FSTL3, CXCL13, PTX3, SMO, KREMEN2, and HOXC10 (**Figures 4B, C**). Multivariable Cox analysis showed that LDHA, COBL, MAPK4, BAIAP2L2, PTX3, and HOXC10 were associated with poorer prognosis in NSCLC (all hazard ratios > 1 and *P* < 0.05), while CXCL13 (hazard ratio = 0.72, *P* = 0.005) was linked to better outcomes (**Figure 4D**). PPI network of these genes highlighted functional interplay in NSCLC pathogenesis (**Figure 4E**), and all these genes exhibited significant differences in gene expression between cancerous and non-cancerous samples (**Figure 4F**).

### Prognostic assessment of T-cell gene signature in TCGA cohorts

**Figure 5:**
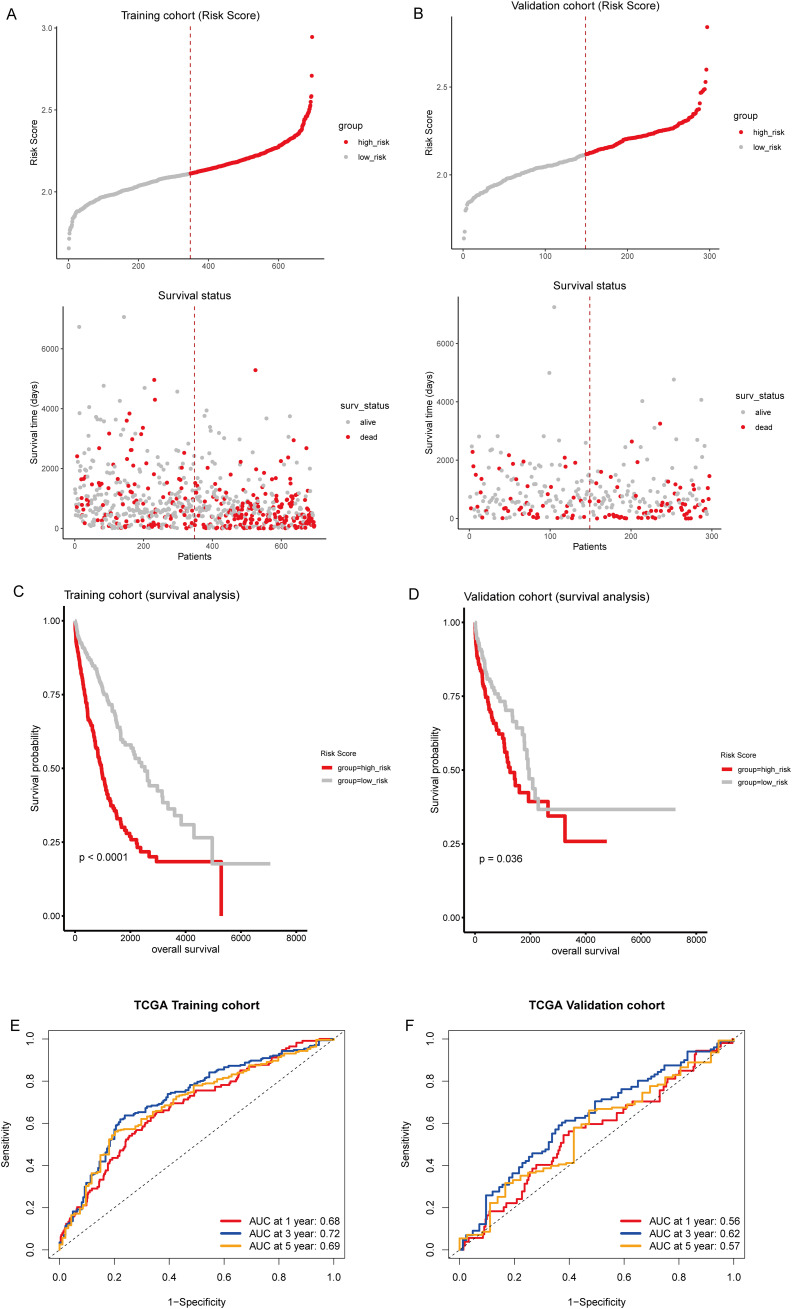
Prognostic evaluation of T-cell-related gene signature. **(A, B)** Upper panel: Risk score distribution in the TCGA training and testing sets, respectively. The dotted line indicates the median risk score threshold for high-risk (red) and low-risk (gray) stratification. Lower panel: Scatter plots correlating individual patient risk scores with survival status. Red dots represent deceased patients, and gray dots represent those who are alive. **(C, D)** Kaplan-Meier survival curves demonstrate the prognosis of high-risk and low-risk patients in the TCGA training and testing sets, respectively. **(E, F)** Time-dependent receiver operating characteristic (ROC) curves for 1, 3, and 5-year overall survival predictions in the TCGA training and testing sets, respectively.

Then, we calculated individual risk scores for each patient by integrating the LASSO coefficients with the expression levels of the 16 genes, according to the formula: (0.102) × LATS2 + (0.483) × LDHA + (0.068) × CKAP4+ (0.036) × COBL + (0.043) × DSG2+ (0.097) × MAPK4 + (0.038) × AKAP12 + (-0.029) × HLF + (-0.045) × CD69 + (0.027) × BAIAP2L2 + (0.111) × FSTL3 + (-0.115) × CXCL13 + (0.055) × PTX3 + (0.044) × SMO + (0.015) × KREMEN2 + (0.001) × HOXC10. Patients from the TCGA training and testing cohorts were stratified into high-risk and low-risk groups based on the median risk score (**Figures 5A, B**). Kaplan-Meier survival analysis confirmed that patients in the high-risk group had significantly poorer prognosis than those in the low-risk group (**Figures 5C, D**). ROC curve analysis demonstrated moderate predictive performance, with area under the curve (AUC) values of 0.68, 0.72, and 0.69 at one, three, and five years, respectively, in the training cohort (**Figure 5E**). In the validation cohort, the AUCs were 0.56, 0.62, and 0.57 at one, three, and five years, respectively (**Figure 5F**). Compared to traditional prognostic factors such as TNM staging and molecular markers like TP53 and Ki-67 (12), the 16-gene model demonstrated moderate predictive accuracy. Integrating clinical parameters may further enhance its prognostic utility.

To further illustrate the distribution of T-cell subsets in these risk strata, we conducted a t-SNE analysis. This analysis revealed a dichotomy between the high-risk and low-risk categories (**Supplementary Figure S1A**). Specifically, Cluster2 exhibited significantly higher risk scores than Cluster1 (**Supplementary Figure S1B**). In the Sankey diagram, a notable portion of the samples from Cluster1 was assigned to the high-risk group, with a consequential flow toward the “Alive” outcome. Conversely, Cluster2 showed a significant representation in the high-risk group, which predominantly correlated with the “Dead” status (**Supplementary Figure S1C**). This visualization effectively highlights the relationship between cluster classification, risk assessment, and survival outcomes.

### Prognostic validation of T-cell gene signature in external GEO cohorts

**Figure 6:**
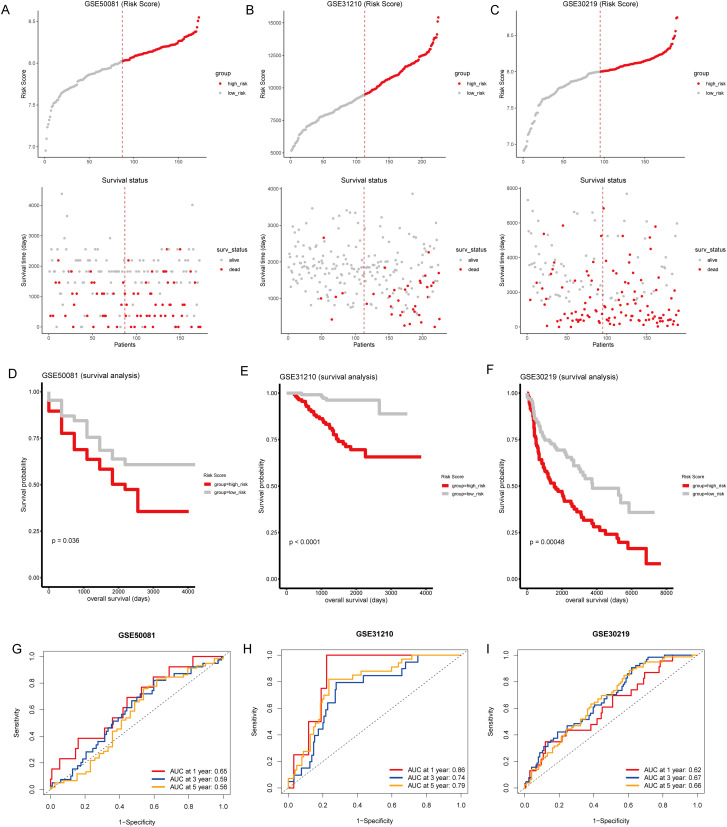
External validation of the T-cell-related gene signature risk score. **(A–C)** Upper panel: Risk score distribution across external GEO cohorts GSE50081, GSE31210, and GSE30219. The dotted line indicates the median risk score threshold for high-risk (red) and low-risk (gray) stratification. Lower panel: Scatter plots correlating individual patient risk scores with survival status. Red dots represent deceased patients, and gray dots represent those who are alive. **(D–F)** Kaplan-Meier survival curves for GSE50081, GSE31210, and GSE30219, respectively, demonstrate significant survival differences between the high-risk and low-risk groups. **(G–I)** Time-dependent ROC curves for 1, 3, and 5-year overall survival predictions in GSE50081, GSE31210, and GSE30219. The area under the curve (AUC) values provide moderate prognostic accuracy across all time points for the three cohorts.

To validate the predictive reliability of the T-cell gene signature, we calculated risk scores for the validation cohorts using the same formula, and samples from external GEO cohorts GSE50081, GSE31210, and GSE30219 were divided into high-risk and low-risk groups (**Figures 6A–C**). We found that patients in the high-risk group had significantly poorer prognosis compared to those in the low-risk group (**Figures 6D–F**). Furthermore, in the external validation sets, the time-dependent ROC curves for predicting 1, 3, and 5-year overall survival based on the risk scores yielded moderate AUC values, with AUCs between 0.56 to 0.59 in GSE50081 (**Figure 6G**), above 0.7 in GSE31210 (**Figure 6H**), and between 0.62 to 0.66 in GSE30219 (**Figure 6I**). These results demonstrate that the T-cell gene signature maintains predictive value across independent patient cohorts, reinforcing its robustness and potential applicability in diverse clinical settings. The variability in AUC values among datasets highlights potential differences in cohort characteristics, sample sizes, and treatment heterogeneity, further underscoring the need for broader validation in prospective studies.

### Clinical significance of the risk score and development of the predictive model

To investigate the clinical significance of the risk score, we stratified TCGA cohorts by age, sex, stage, and TNM classification. Across these subgroups, high-risk groups consistently exhibited significantly poorer survival compared to their low-risk counterparts (all *P* < 0.001; **Supplementary Figures S2, S3**). Tumors with higher TNM classifications (M1, T3-T4, N1-N3) tended to have elevated risk scores compared to their lower TNM counterparts (M0, T1-T2, N0) (all *P* < 0.05; **Supplementary Figure S3G**).

**Figure 7:**
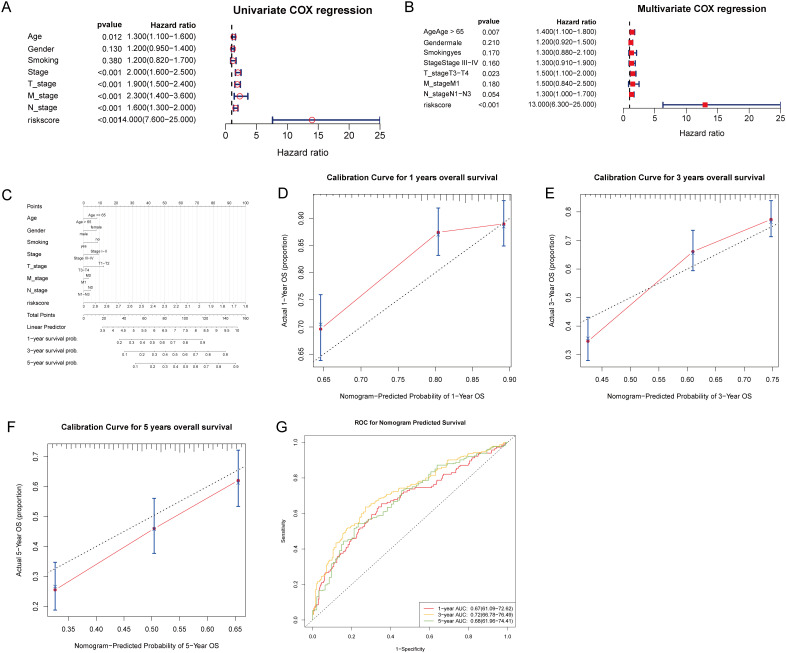
Prognostic evaluation of T-cell gene signature risk score with clinical parameters in patient survival analysis. **(A)** Univariate Cox regression analysis was performed to evaluate the association of clinical factors and the risk score with patient survival. **(B)** Multivariate Cox regression analysis identified the risk score, age > 65, and T3-T4 stage as independent prognostic factors. **(C)** A nomogram incorporating the risk score and clinical factors was created to predict 1, 3, and 5-year patient survival probabilities, with total points corresponding to predicted outcomes. **(D–F)** Calibration curves for the nomogram’s 1, 3, and 5-year overall survival predictions. **(G)** ROC curves were generated to evaluate the nomogram’s predictive accuracy for 1, 3, and 5-year overall survival, with AUC values indicating the model’s discriminative ability.

Subsequently, we conducted univariate and multivariate Cox regression analyses to identify independent prognostic clinical factors. Univariate Cox regression analysis revealed significant associations between the risk score, age, stage, and TNM classification with patient survival (all *P* < 0.05; **Figure 7A**). Multivariate Cox regression confirmed the risk score as an independent prognostic factor (*P* < 0.001), along with age > 65 (*P* = 0.007) and T3-T4 classification (*P* = 0.023; **Figure 7B**). Then, we created a nomogram that incorporates the risk score and clinical factors (age, sex, smoking status, stage, TNM classification) to predict 1, 3, and 5-year survival probabilities for patients (**Figure 7C**). Calibration plots showed strong agreement between observed and nomogram-predicted survival probabilities at each time point (**Figures 7D–F**). Furthermore, the ROC curve analysis for 1, 3, and 5 years confirmed the model’s robust predictive accuracy for clinical application (all AUCs > 0.6; **Figure 7G**).

### T-cell-related risk score is associated with tumor immune microenvironment in NSCLC

**Figure 8:**
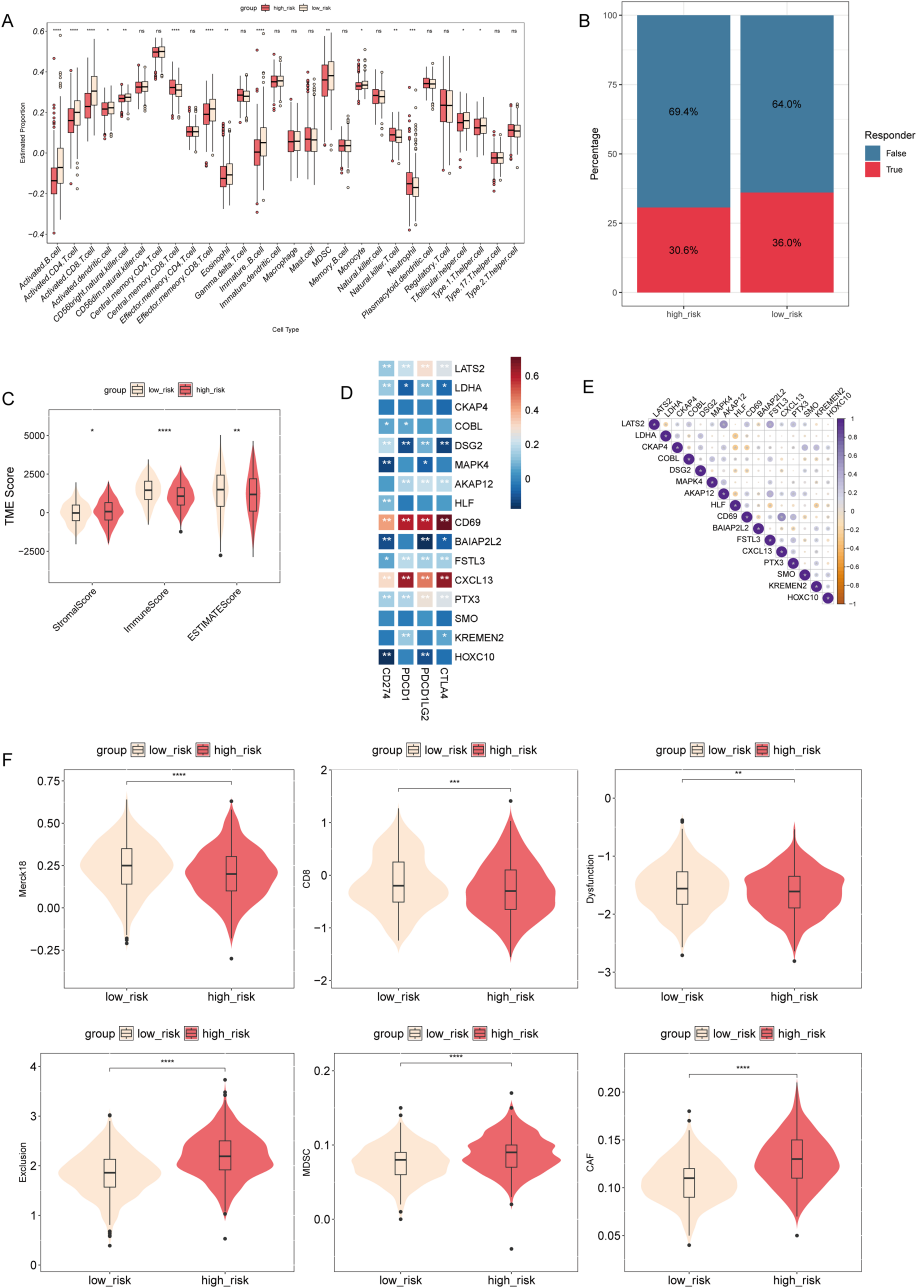
Association between risk score and tumor immune microenvironment in NSCLC. **(A)** Comparison of the levels of immune cell infiltration in the tumor microenvironment between high- and low-risk groups. ^*^ *P* < 0.05, ^**^ *P* < 0.01, ^***^ *P* < 0.001, ^****^ *P* < 0.0001. **(B)** TIDE algorithm analysis was performed to differentiate between the immune therapy responders and non-responders in different risk groups. **(C)** Comparison of stromal, immune, and ESTIMATE scores between high- and low-risk groups. ^*^ *P* < 0.05, ^**^ *P* < 0.01, ^****^ *P* < 0.0001. **(D)** A heatmap was generated to show the correlation between model genes and immune checkpoint genes. **(E)** Correlation matrix plotting was performed to visualize the pairwise correlation coefficients among model genes. **(F)** Comparison of TIDE scores between different risk groups. ns, not statistically significant (*p* ≥ 0.05).

To investigate the potential relationship between risk score and the tumor immune microenvironment, we utilized the ssGSEA algorithm to assess immune cell infiltration levels and compared them with the risk score. We observed significant differences in 15 immune cell types, with elevated levels of central memory CD8 T-cells, natural killer T-cells, and neutrophils notably higher in the high-risk group (**Figure 8A**). Furthermore, the TIDE algorithm revealed a lower proportion of immune therapy responders among patients with high-risk scores compared to non-responders (**Figure 8B**). Evaluation using the ESTIMATE algorithm unveiled notably higher stromal scores in the high-risk group, while immune scores and ESTIMATE scores were significantly greater in the low-risk group (**Figure 8C**). Correlation analysis identified varying associations between model genes and immune checkpoint genes, as well as among model genes (**Figures 8D, E**). The distribution of TIDE scores highlighted significantly elevated T-cell exclusion, myeloid-derived suppressor cells (MDSCs), and cancer-associated fibroblasts (CAFs) in the high-risk group, while immune checkpoints and T-cell dysfunction scores were reduced compared to the low-risk group (**Figure 8F**). The results of the GSEA showed that, among the Hallmark pathways, there were notable enrichments in “epithelial mesenchymal transition”, “hypoxia”, “IL2 STAT5 signaling”, “inflammatory response”, “KRAS signaling up”, “MTORC1 signaling”, “P53 pathway”, and “TNFA signaling via NFKB” in the high-risk group (**Supplementary Figure S4**, **Supplementary Table S6**). These data suggest that high-risk patients may exhibit augmented immune cell infiltration levels, decreased response to immune therapy, and alterations in immune checkpoint expression compared to low-risk patients.

### Differential drug sensitivity prediction in high- and low-risk patients

To evaluate drug sensitivity among patients at different risk levels, we employed the “oncoPredict” R package. Our analysis revealed significant differences in drug responses between high- and low-risk groups (**Supplementary Figures S5A–F**). Furthermore, these distinctions in drug responsiveness correlated with risk scores (**Supplementary Figures S5G–L**). For example, AZD5991-1720 exhibited enhanced response in high-risk patients, positively correlating with risk score (**Supplementary Figures S5D, J**). Conversely, IGF1R-3801-1738 demonstrated improved response in the low-risk group, negatively correlating with risk score (**Supplementary Figures S5E, K**). Complete drug prediction results are provided in **Supplementary Table S7**.

### Validation of model gene alteration in clinical NSCLC samples

**Figure 9:**
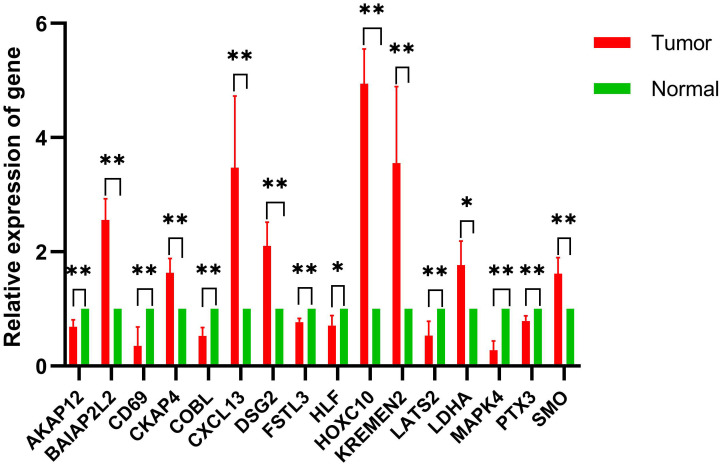
Comparative analysis of gene expression levels between control and tumor samples. qRT-PCR was performed to determine model gene expression in NSCLC tumor samples and control samples. *p < 0.05, **p < 0.01; n = 4.

To validate model gene alterations in clinical NSCLC samples, we compared the relative expression levels of these genes between tumor and adjacent normal tissues from four NSCLC patients. The results of the qRT-PCR analysis showed that BAIAP2L2, CKAP4, CXCL13, DSG2, HOXC10, KREMEN2, LDHA, and SMO were upregulated in tumor samples compared to normal tissue. Conversely, AKAP12, CD69, COBL, FSTL3, HLF, LATS2, MAPK4, and PTX3 were downregulated in tumor samples relative to normal tissue (**Figure 9**). These findings are consistent with the expression patterns observed in the TCGA cohorts.

## Discussion

In this study, we investigated the role of T-cell-related genes in non-small cell lung cancer (NSCLC) and their potential implications for prognosis and treatment strategies. Through integrative analysis of large-scale transcriptomic data from TCGA, we identified T-cell-related DEGs and characterized T-cell-related subtypes in NSCLC. Additionally, we developed a 16-gene prognostic signature based on T-cell-related genes and validated its predictive power in both internal and external cohorts. Our analysis also revealed significant associations between the T-cell-related risk score and the tumor immune microenvironment, as well as differential drug sensitivities between high- and low-risk patients. Validation of gene expression in clinical samples showed that all 16 model genes were differentially expressed between tumor and normal tissues, consistent with trends observed in the TCGA dataset. These findings provide valuable insights into the role of T-cell-related genes in NSCLC pathogenesis, prognosis, and potential therapeutic strategies.

This study identified a 16-gene signature that is closely linked to NSCLC prognosis and immune microenvironment composition. Key genes include LDHA, MAPK4, HOXC10, and CXCL13, each of which plays a role in critical processes such as metabolism, signal transduction, and immune response. LDHA, known for its role in glycolysis under hypoxia, is overexpressed in LUAD and associated with poor outcomes (34). Inhibiting LDHA has been shown to enhance T-cell-mediated immunity by reducing lactate buildup in the tumor microenvironment (35). Unlike other identified biomarkers, CXCL13 showed a positive association with improved outcomes. This chemokine promotes immune cell recruitment and the formation of tertiary lymphoid structures, which enhance antitumor immunity and improve survival in NSCLC (36–38). This distinction highlights the dual role of the immune microenvironment in cancer, in which CXCL13 enhances immune surveillance, while LDHA contributes to immune evasion. Lactate from LDHA-overexpressing tumors may suppress CXCL13-mediated immune cell recruitment, creating a “cold” tumor microenvironment. Targeting LDHA could reduce lactate accumulation, potentially synergizing with CXCL13-boosting strategies to convert “cold” tumors into “hot” ones, enhancing immune responses and ICI efficacy. This dual approach warrants experimental validation in preclinical models.

Other genes, including MAPK4 and HOXC10, further illustrate the diverse roles of T-cell-related genes in NSCLC progression. MAPK4, a kinase involved in cellular signal transduction, has been linked to angiogenesis and tumor growth, with higher expression correlating with poor prognosis (39). Similarly, HOXC10, a transcription factor, promotes tumor progression by inducing cell proliferation and inhibiting apoptosis (40, 41). It also facilitates immune evasion by upregulating immunosuppressive factors, such as PD-L2, which inhibit T-cell-mediated tumor clearance (42, 43). These genes are not only potential prognostic biomarkers but also represent potential therapeutic targets, offering opportunities for more effective immunotherapy approaches in NSCLC.

The identified 16-gene T-cell-related signature offers substantial clinical value by stratifying NSCLC patients into high- and low-risk groups, each with distinct survival outcomes and therapeutic responses. High-risk patients, characterized by elevated expression of genes like LDHA and HOXC10, showed significantly poorer survival and reduced responsiveness to immune checkpoint inhibitors (ICIs) targeting PD-1/PD-L1. In contrast, low-risk patients with higher expression of CXCL13 were associated with better survival and potentially enhanced response to ICIs. This stratification allows for more personalized therapeutic approaches, highlighting those more likely to benefit from immunotherapy. Moreover, the relationship between gene expression and drug sensitivity enhances the clinical utility of this signature. High-risk patients showed greater sensitivity to AZD5991, an MCL1 inhibitor, which targets apoptosis resistance driven by metabolic stress and immune suppression (44), suggesting that these patients may benefit from combining MCL1 inhibitors with checkpoint blockade to counteract T-cell exhaustion. In contrast, low-risk patients responded better to IGF1R inhibitors, possibly due to IGF1R’s role in maintaining tertiary lymphoid structures and activating T-cells (45). These patients might benefit significantly from immunotherapy (e.g., anti-PD-1), supported by their higher immune scores. This differentiation suggests tailored therapeutic pathways based on patient risk profiles.

Furthermore, classifying NSCLC patients into distinct T-cell-related subtypes has significant clinical implications. High-risk patients with increased infiltration of immunosuppressive cells (e.g., MDSCs, CAFs) may benefit from combination therapies incorporating immune-modulating agents alongside ICIs to overcome resistance. Conversely, low-risk patients with enhanced immune activation may respond favorably to ICIs alone or immune-stimulatory interventions. These findings support a tailored approach to immunotherapy selection, optimizing treatment strategies based on immune subtype classification. By integrating the 16-gene signature with existing biomarkers, such as PD-L1, clinical decision-making can be refined. High-risk patients with elevated LDHA may be prioritized for metabolic-targeted therapies (e.g., LDHA inhibitors combined with chemotherapy), while low-risk patients with elevated CXCL13 could be directed toward immunotherapy. These findings support a tailored approach to treatment selection, enhancing the effectiveness of personalized therapies in NSCLC. Therefore, the 16-gene signature serves not only as a prognostic tool but also as a guide for optimizing and personalizing treatment strategies in NSCLC.

Our study adds to the understanding of T-cells and immune-related biomarkers in NSCLC. Previous investigations have often focused on the predictive value of PD-1/PD-L1 expression for immunotherapy responses, our approach extends beyond these conventional markers. By identifying a broader panel of T-cell-related genes, we provided deeper insights into how these genes influence both prognosis and immune responses in NSCLC. Notably, genes such as COBL (involved in cytoskeletal organization) (46) and FSTL3 (linked to immune cell infiltration) (47) were included in our analysis, underscoring the complexity and heterogeneity of the immune microenvironment in NSCLC. This comprehensive perspective provides a deeper understanding of the varied roles of T-cell biology in influencing patient outcomes and therapeutic responses. Additionally, our findings complement recent studies highlighting the importance of the tumor immune microenvironment in shaping NSCLC treatment outcomes. For instance, BAIAP2L2 was found to negatively correlate with immune checkpoint expression, suggesting that certain genes may actively suppress immune responses, facilitating immune evasion by tumors. This aligns with the growing recognition of the need for combination therapies that target both immune escape mechanisms and traditional oncogenic pathways (48), which could be explored in future research.

Despite these promising results, our study has limitations. First, we relied on retrospective data from publicly available datasets, which may introduce selection bias and limit the generalizability of our findings. Although we validated our model in multiple external cohorts, prospective clinical validation is necessary to confirm its utility in real-world clinical settings. Additionally, while our model demonstrated robust performance in predicting patient outcomes, the underlying biological mechanisms linking these genes to NSCLC prognosis and immunotherapy response require further investigation.

Future studies should focus on validating our 16-gene signature in larger, multi-center clinical trials to assess its predictive value across diverse patient populations. Investigating the functional roles of the identified genes in modulating immune responses in NSCLC through *in vitro* and *in vivo* experiments would also be valuable. Moreover, given the dynamic nature of the tumor immune microenvironment, longitudinal studies assessing gene expression changes over time and in response to treatment would provide a deeper understanding of how T-cell-related genes impact disease progression and therapeutic outcomes.

## Conclusion

In conclusion, our study identified distinct T-cell-related subtypes in NSCLC and developed a robust prognostic gene signature. These findings provide insights into the immune microenvironment and provide a potential tool for patient risk stratification and treatment planning. The 16-gene signature can be used to stratify NSCLC patients into high-risk and low-risk groups, guiding treatment decisions such as using immune checkpoint inhibitors, chemotherapy, or targeted therapies. Furthermore, incorporating this gene signature into clinical practice could help identify patients most likely to benefit from personalized immunotherapies, potentially improving survival outcomes. Integrating this signature into clinical decision support systems could enhance oncologists’ ability to make informed, data-driven treatment choices based on a patient’s genetic and immune profile. However, further prospective validation and clinical studies are necessary to fully realize the clinical implications of these results and their potential role in personalized treatment strategies for NSCLC.

## Data Availability

The raw data supporting the conclusions of this article will be made available by the authors, without undue reservation.
