# Supplementary material for: Development and validation of a 16-gene T-cell- related prognostic model in non-small cell lung cancer
Source: Front Immunol. 2025 Apr 7;16:1566597. doi: 10.3389/fimmu.2025.1566597 (PMC12009871; doi:10.3389/fimmu.2025.1566597)
Supplement: Supplementary file 7 [file SupplementaryFile1.docx]

**Figure S1: T-cell subsets stratification and prognostic correlation in risk groups.** (A) A t-distributed stochastic neighbor embedding (t-SNE) analysis was performed to illustrate the distribution of T-cell subsets in high-risk (yellow) and low-risk (blue) groups in the TCGA cohorts. (B) Comparison of the risk scores between T-cell clusters. ^****^*P* < 0.0001. (C) A Sankey diagram was generated to visualize the flow of patient samples from two T-cell clusters into high and low-risk categories, and their subsequent status as "Alive" or "Dead". The widths of the bands correspond to the number of samples transitioning between states.

**Figure S2: Kaplan-Meier survival analysis by age, sex, and stage subgroups in TCGA cohorts.** Patients were stratified by age (A, B), sex (C, D), and stage (E, F) to evaluate the prognostic validity of the T-cell-related gene signature risk score.

**Figure S3: Kaplan-Meier survival analysis by T, M, and N classifications in TCGA cohorts.** Patients were stratified by T (A, B), M (C, D), and N (E, F) classifications to evaluate the prognostic validity of the T-cell-related gene signature risk score. (G) Tumors with higher TNM classifications (M1, T3-T4, N1-N3) tended to have elevated risk scores compared to their lower TNM counterparts (M0, T1-T2, N0). T, tumor; N, nodes; M, metastasis.

**Figure S4: Gene set enrichment analysis of hallmark pathways in TCGA patient cohort categorized by median risk score.**

**Figure S5: Prediction of drug sensitivity in high- and low-risk patient groups using oncoPredict.** (A-F) Comparison of the predicted sensitivity scores for different drugs between low-risk and high-risk patient groups. (G-L) Scatter plots illustrate the correlation between risk scores and drug sensitivity. Each point represents a patient's risk score plotted against their sensitivity to a specific drug, with regression lines indicating the trend.

Table S1: Primers for qRT-PCR.

Table S2: List of DEGs in cancer tissue samples compared to non-cancerous tissue samples in the TCGA NSCLC cohorts.

Table S3: Gene Ontology enrichment analysis for DEGs.

Table S4: Differential Gene Ontology terms across patient subtypes.

Table S5: Differential KEGG pathways across patient subtypes.

Table S6: GSEA Hallmark pathway enrichment in high-risk versus low-risk TCGA patient groups.

Table S7: oncoPredict drug sensitivity results
