# Supplementary material for: Development and validation of a 16-gene T-cell- related prognostic model in non-small cell lung cancer
Source: Front Immunol. 2025 Apr 7;16:1566597. doi: 10.3389/fimmu.2025.1566597 (PMC12009871; doi:10.3389/fimmu.2025.1566597)
Supplement: Supplementary file 8 [file Table1.docx]

Table S1 Primers for qRT-PCR.

| **Gene name** | **Base sequence** |
| --- | --- |
| HOXC10-F | ACATGCCCTCGCAATGTAACT |
| HOXC10-R | GAGAGGTAGGACGGATAGGTG |
| KREMEN2-F | AGGGCATCTACTGGCGCTA |
| KREMEN2-R | CTGAGTCCACAAAGCATCCCA |
| SMO-F | CTGTCCTGCGTCATCATCTTT |
| SMO-R | CCACAGCAAGGATTGCCAC |
| PTX3-F | TTATTCCCAATGCGTTCCAAGA |
| PTX3-R | GCACTAAAAGACTCAAGCCTCAT |
| CXCL13-F | GCTTGAGGTGTAGATGTGTCC |
| CXCL13-R | CCCACGGGGCAAGATTTGAA |
| FSTL3-F | GTGCCTCCGGCAACATTGA |
| FSTL3-R | GCACGAATCTTTGCAGGGA |
| BAIAP2L2-F | CTCCATAGCACCCTCGGAGTA |
| BAIAP2L2-R | GCAAAAGGATTTGTGCCCCTC |
| CD69-F | ATTGTCCAGGCCAATACACATT |
| CD69-R | CCTCTCTACCTGCGTATCGTTTT |
| HLF-F | CTGGGGCCTACCTTATGGGA |
| HLF-R | GGGGAATGCCATTTTCTGACA |
| AKAP12-F | ATCTACAGAGAAACCCGAAGAGA |
| AKAP12-R | TGCAGACTTGCTAGGTTCTTTTT |
| MAPK4-F | ACCACGACAACATCGTCAAAG |
| MAPK4-R | TACGCCACGCTGAACTTGAA |
| DSG2-F | TTGTTGGGTCTGTTGAAGAGTTG |
| DSG2-R | TTCAGGGTATTGGGCTCATCT |
| CKAP4-F | TGGACAGTTTGGTTGCATACTC |
| CKAP4-R | CCTCAGGTCATCTAGTAAACCCT |
| LDHA-F | TTGACCTACGTGGCTTGGAAG |
| LDHA-R | GGTAACGGAATCGGGCTGAAT |
| LATS2-F | ACTTTTCCTGCCACGACTTATTC |
| LATS2-R | GATGGCTGTTTTAACCCCTCA |
| COBL-F | GTGCGTTTGGTCGTGAATTAC |
| COBL-R | GACCTCACACTTTGCACAAATG |
| GAPDH-F | AGATCCCTCCAAAATCAAGTGG |
| GAPDH-R | GGCAGAGATGATGACCCTTTT |
