# Supplementary figures and images for: Development and validation of a 16-gene T-cell- related prognostic model in non-small cell lung cancer
Source: Front Immunol. 2025 Apr 7;16:1566597. doi: 10.3389/fimmu.2025.1566597 (PMC12009871; doi:10.3389/fimmu.2025.1566597)

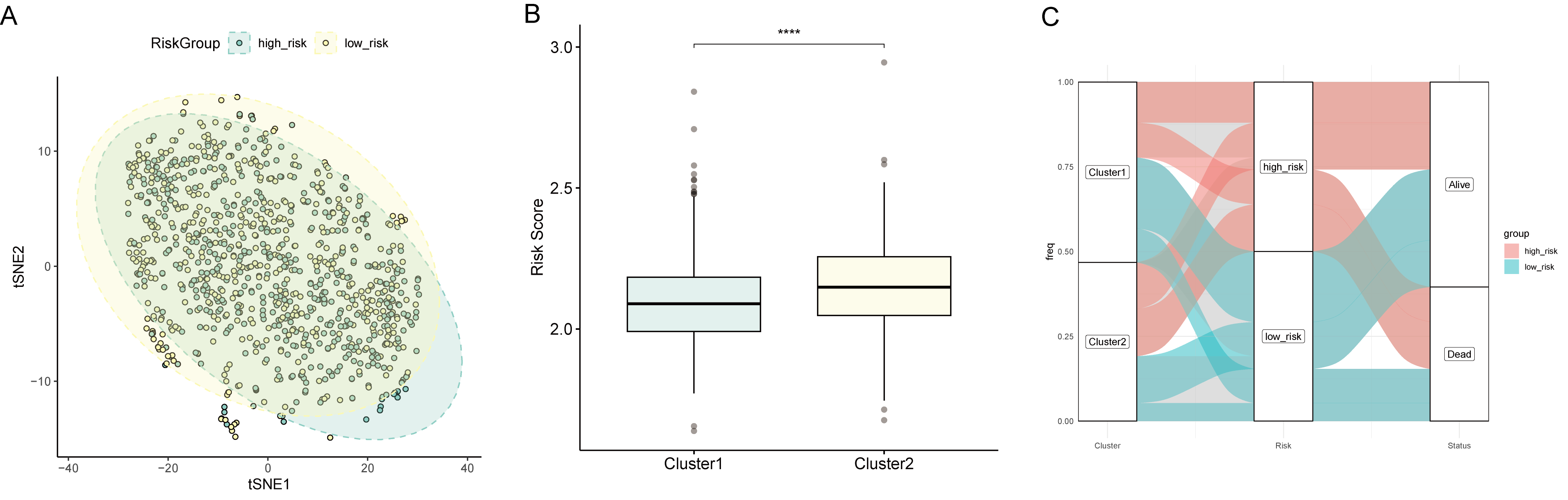

Supplement: Supplementary file 9 [file Image1.tif]

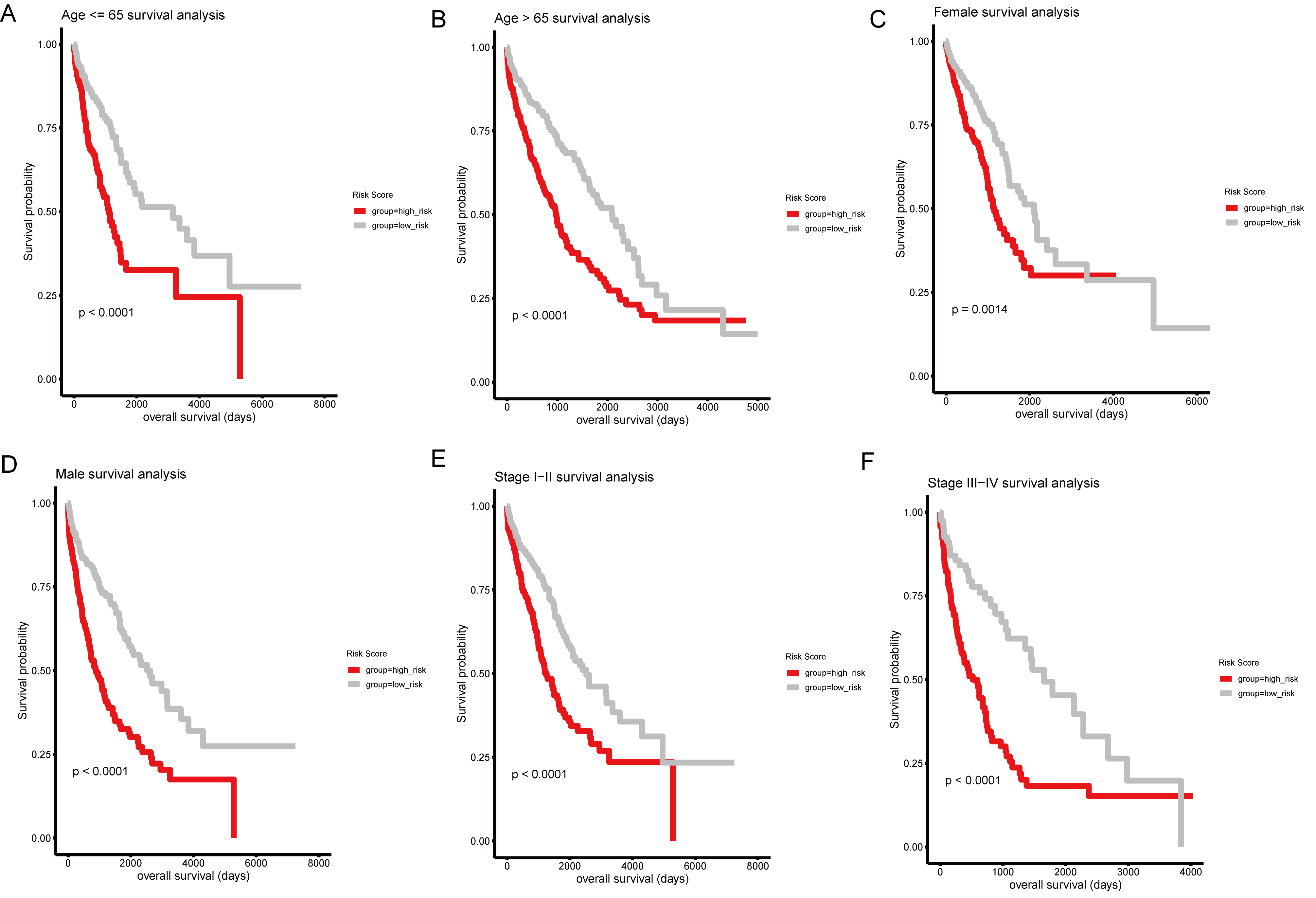

Supplement: Supplementary file 10 [file Image2.tif]

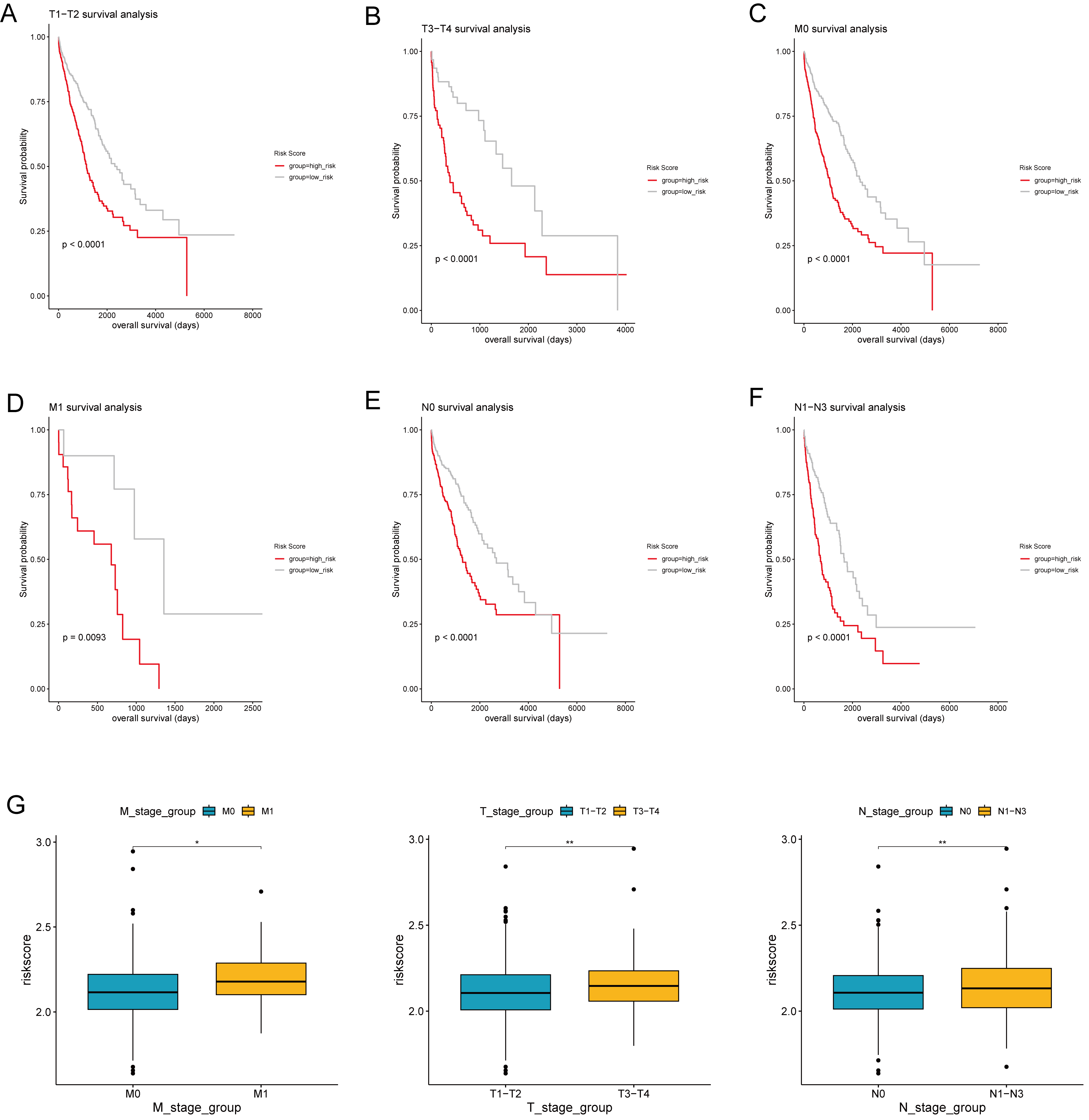

Supplement: Supplementary file 11 [file Image3.tif]

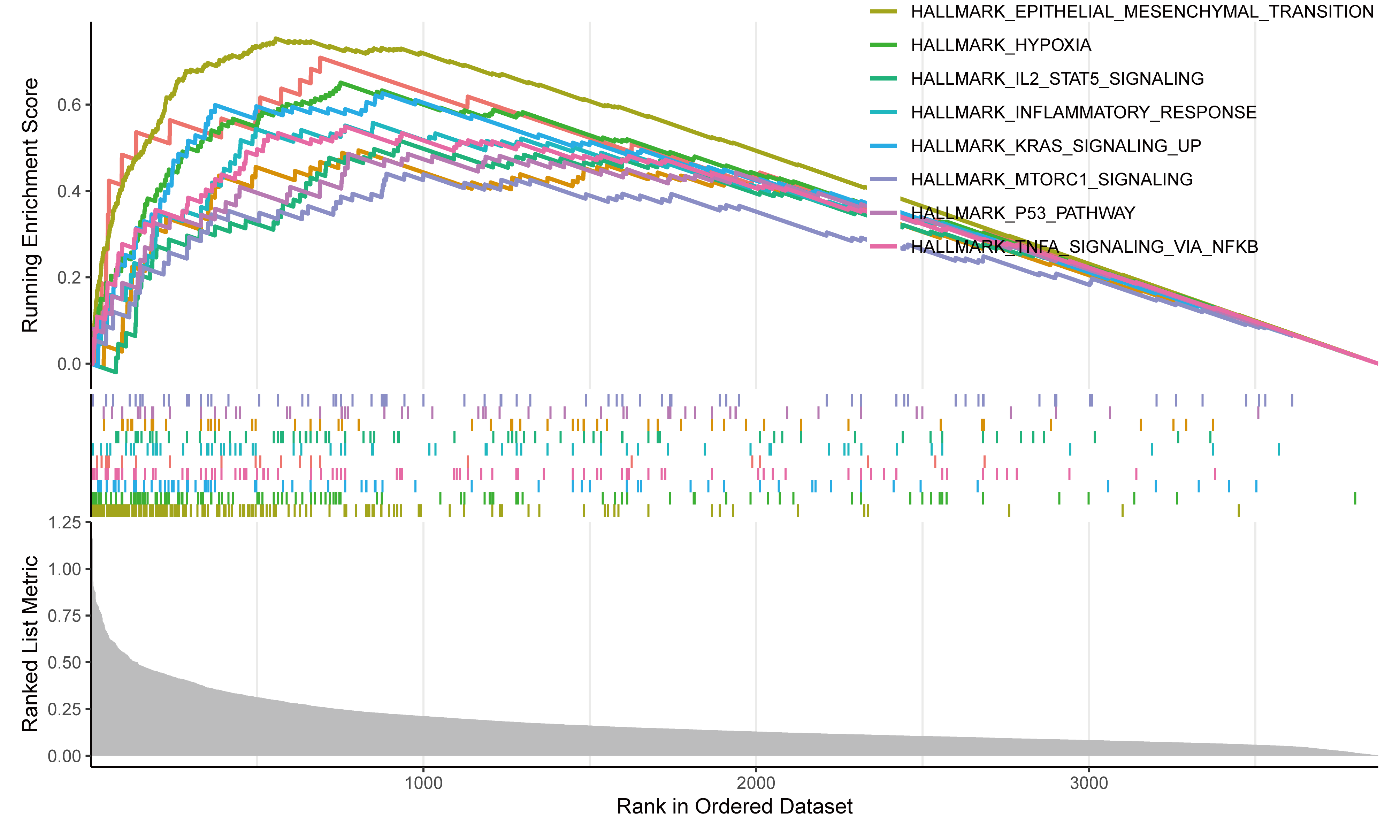

Supplement: Supplementary file 12 [file Image4.tif]

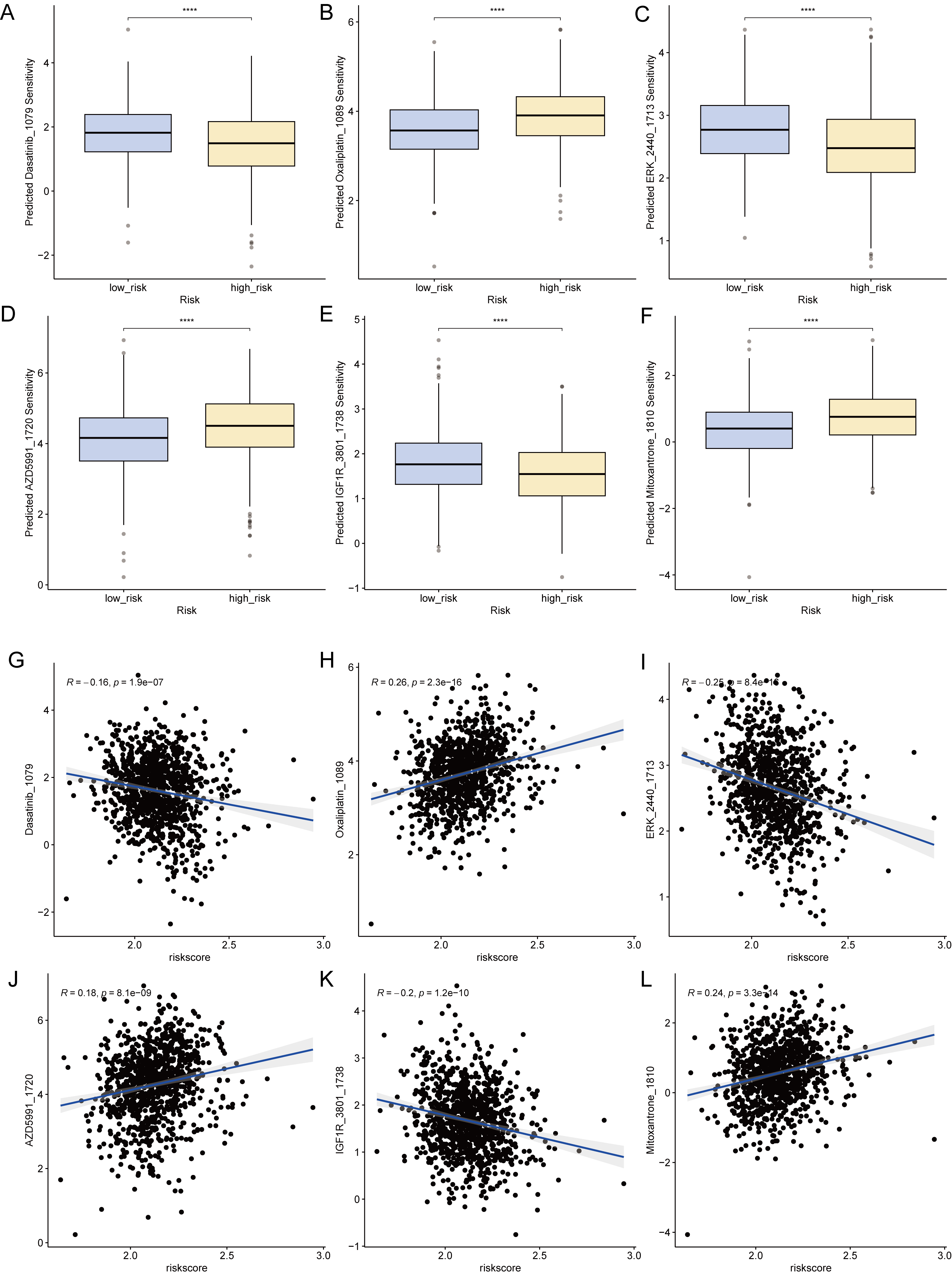

Supplement: Supplementary file 13 [file Image5.tif]
